# Online Crowdsourced Data from iNaturalist Can Assist Monitoring of Invasive Mosquitoes

**DOI:** 10.3390/insects16020128

**Published:** 2025-01-28

**Authors:** Benjamin Cull

**Affiliations:** Department of Entomology, College of Food, Agricultural and Natural Resource Sciences, University of Minnesota, St. Paul, MN 55108, USA; cull0122@umn.edu

**Keywords:** vector surveillance, *Aedes aegypti*, *Aedes albopictus*, *Aedes japonicus*, *Aedes koreicus*, invasive species, community science, citizen science, mosquito

## Abstract

Invasive mosquitoes continue to spread, increasing the threat of mosquito-borne disease. Ongoing mosquito surveillance is necessary to track the introduction and establishment of these species in new areas and implement appropriate public health and vector control measures. Contributions from citizen science initiatives have been an important component in detecting, controlling, and raising awareness of invasive mosquitoes. The open-access biodiversity platform iNaturalist is an extensive source of human observations of wildlife, including arthropod vectors, and can be a useful supplementary tool for passive vector surveillance. In this study, the utility of iNaturalist data to support invasive mosquito surveillance was assessed by examining the distribution and seasonal data on four invasive *Aedes* species (IAS) in Europe and neighbouring countries. Almost 16,000 iNaturalist observations of mosquitoes were examined across 62 countries; 13% were identified as IAS, with a further 2% considered probable IAS. These included 16 observations of *Aedes aegypti*, 1582 *Aedes albopictus*, 373 *Aedes japonicus,* and 58 *Aedes koreicus.* iNaturalist observations of IAS were present in most known areas of establishment, but potential new regions of spread were also identified. These results further support the use of iNaturalist data as a low-cost source of arthropod data to assist existing vector surveillance.

## Simple Summary

Invasive mosquitoes, such as the Asian tiger mosquito *Aedes albopictus*, continue to spread, increasing the risk of mosquito-borne disease. The establishment of this mosquito in Europe has enabled outbreaks of chikungunya, dengue, and Zika, previously considered tropical diseases. The development of low-cost and effective surveillance methods is necessary to enable sustainable monitoring of vector distributions to detect and control populations of invasive mosquitoes. Previous work has shown the potential of iNaturalist, an open-access, online, community-populated biodiversity recording database, to be valuable for the surveillance of important arthropod vectors. In this study, iNaturalist data is applied to the surveillance of invasive mosquitoes across Europe and neighbouring countries and compared with data from the VectorNet project, which monitors the distribution of important arthropod vectors across Europe. The results show that iNaturalist data generally match the known distribution and seasonal activity of invasive mosquitoes across the studied region, and importantly, iNaturalist data recorded invasive mosquitoes in multiple areas without existing records, showing that iNaturalist has the potential for identifying new areas of expansion that could be targeted with further surveillance and control strategies.

## 1. Introduction

The establishment and spread of non-native or invasive arthropod vector species can lead to the introduction of associated vector-borne pathogens, leading to increased disease risk in invaded areas. For example, the recent introduction and expansion of the Asian long-horned tick *Haemaphysalis longicornis* in the United States has been associated with outbreaks of theileriosis in infested cattle [1,2]. Similarly, the establishment of invasive *Aedes aegypti* and *Aedes albopictus* mosquitoes has led to locally transmitted outbreaks of chikungunya, dengue, and Zika in multiple European countries in recent years [3,4]. These invasive mosquitoes represent a considerable public health and economic burden [5]. In addition to *Ae. albopictus*, which has continued to spread throughout continental Europe since its introduction in 1990, two other invasive mosquitoes, *Aedes japonicus* and *Aedes koreicus*, have also become established [6]. These mosquitoes also present a disease threat, as *Ae. japonicus* has been shown to be competent in transmitting a number of arboviruses, among them chikungunya, dengue, and West Nile viruses [7,8,9], whilst *Ae. koreicus* is considered a competent vector for the chikungunya virus and the heartworm *Dirofilaria immitis* [10]. Ongoing integrated surveillance programs to monitor the distribution and activity of these invasive species are essential for controlling their spread and limiting the associated risks from mosquito-borne disease.

Community science initiatives involving the submission of mosquito specimens or photos by the public have been useful tools for invasive mosquito surveillance, as well as for raising public awareness of these insects and household-level control measures [11,12,13,14]. Major benefits of these programs include their low cost and wide coverage relative to more labor-intensive active surveillance methods. In many cases these projects have identified new areas of invasive mosquito introduction, allowing enhanced surveillance by local authorities [15,16]. Smartphone application-based surveillance tools, such as Mosquito Alert [12,17] and GLOBE Observer Mosquito Habitat Mapper [18], are a convenient way for community members to record and contribute information that can be rapidly transmitted to researchers and vector control professionals who can utilise the data. General biodiversity recording applications such as iNaturalist are also promising methods for collecting data on arthropod vectors [19,20,21]. Compiling data from multiple such community science platforms may be considered the next generation of vector surveillance [22], which would provide additional information to support existing surveillance and control on the ground.

It was recently shown that mosquito observations from iNaturalist provided information on species composition, distribution, and seasonal activity comparable to other data sources [23]. That analysis also confirmed that most mosquito observations took place in urban areas and were of species that used oviposition sites close to human dwellings–unsurprising given that these data consist primarily of opportunistic human sightings of mosquitoes. However, it was concluded that these features would lend support to the use of iNaturalist for invasive mosquito species detection, given that these species show a tendency to breed in manmade containers in urban areas and exhibit anthropophilic feeding tendencies [24,25].

Therefore, this study aimed to assess how well mosquito data from iNaturalist represented the known distribution of four invasive mosquito species (*Ae. aegypti*, *Ae. albopictus*, *Ae. japonicus,* and *Ae. koreicus*) in Europe and neighbouring countries. Mosquito observations from iNaturalist were identified, and their distribution was compared to invasive mosquito distribution data from the European VectorNet project. This joint project of the European Centre for Disease Prevention and Control (ECDC) and the European Food Safety Authority (EFSA) oversees the collection, mapping, and sharing of distribution data of arthropod vectors of animal and health concern [26] and provides regularly updated regional maps of vector species occurrence across the European continent [27]. The analysis reported here found that iNaturalist data on the four mosquito species studied provided distribution information representative of VectorNet maps (i.e., known species distribution) but under-recorded species distribution at a regional level. However, iNaturalist observations of invasive mosquitoes were identified in regional units of multiple countries that do not have existing VectorNet records, suggesting that iNaturalist can support existing surveillance by helping to identify new areas of invasive mosquito expansion.

## 2. Materials and Methods

### 2.1. Data Acquisition

iNaturalist is an online platform where anybody can share biodiversity information, primarily achieved through uploading photos of wildlife captured using a smartphone camera through the iNaturalist app. These “observations” include date and location information. Upon uploading an image, iNaturalist provides suggested identifications based on similarity to identified images in its database and the observation’s geographic location, and users are also free to suggest their own identification. The identification of observations uses a crowd-sourced identification system in which the identification is fine-tuned by other iNaturalist users. Users encompass a broad range of experience levels, from amateur naturalists interested in identifying wildlife seen around their home to experienced scientists collecting biodiversity information. In terms of mosquito observations, this means that no knowledge of mosquitoes is required to record a mosquito observation, and the photographic quality and identification accuracy could vary widely. Therefore, in this study, a broad search of all Culicidae observations was employed, followed by expert validation, to detect both identified IAS observations as well as those that may not have been identified at the species level.

Mosquito observations were downloaded from iNaturalist (iNaturalist.org (accessed on 8 October 2024)) on a country-by-country basis during 2024 by filtering data by taxon Culicidae and country and downloading all observations until the end of 2023. Images associated with observations were identified as far as possible with the aid of keys to European mosquitoes [28]. *Aedes japonicus* and *Aedes koreicus* were differentiated using the characteristics defined by Pfitzner et al. [29], (i.e., the base of the hind femur [completely pale-scaled in *Ae. koreicus* and with a dark sub-basal band in *Ae. japonicus*]), and banding of hind tarsi; if the image did not include sufficient detail to separate the species, the observation was identified as *Aedes japonicus*/*koreicus*. Identification of invasive mosquitoes was categorized as ‘confirmed’ and ‘suspected’ as previously [19], (i.e., if all features necessary for identification were visible, observations were classified as ‘confirmed’, but if only partial characteristics were visible, identification was ‘suspected’). Data were cleaned in Microsoft Excel by removing data fields that were not relevant to the study. Raw, cleaned data used in this study can be found in Table S1.

### 2.2. Mapping

Data were mapped and analysed using ArcGIS online, version 2024.3 (ESRI). Point data from iNaturalist Culicidae observations (Table S1) were mapped and joined to layers of Nomenclature of Territorial Units for Statistics (NUTS) regions level 2 and 3 (or equivalent) to show the regional distribution of IAS. Geographical occurrences of IAS observed on iNaturalist to the end of 2023 were then compared at the equivalent NUTS2/3 regional scale to the VectorNet project’s mosquito maps published in October 2023 [30] by recording the presence or absence of IAS in each regional unit for each dataset.

### 2.3. Seasonal Occurrence of Mosquito Observations

The month of observation was used to examine the seasonality of human encounters with IAS. Due to a wide variation in year-to-year observations, partly due to the rapidly increasing use of iNaturalist in recent years, data were expressed as the mean proportion of mosquito observations per month during 2020–2023, as there were few mosquito observations prior to 2020. The mean proportion of observations per month and the 95% confidence intervals were calculated and plotted using GraphPad Prism 10 software.

## 3. Results

### 3.1. Invasive Aedes Observations

**Figure 1:**
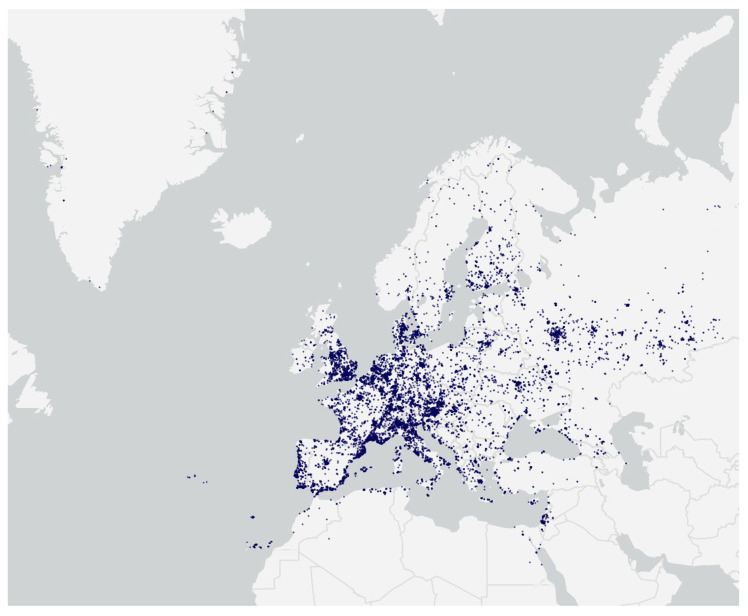
Distribution of iNaturalist observations of Culicidae in Europe and neighbouring countries to the end of 2023.

A total of 15,835 observations of Culicidae were examined from across Europe and neighbouring countries (Figure 1), and 2427 (15.3%) of these were identified as confirmed (2054; 13.0%) or suspected (373; 2.4%) invasive *Aedes* species (Table 1 and Table S2). Observations of IAS were identified in 37 (59.7%) of the 62 countries investigated. For many countries (14/37; 37.8%) with identified observations of IAS, these species constituted over 20% of all mosquito observations, and in nine countries, IAS observations made up over 30% of all mosquito observations (Table 1). The majority of IAS observations (91.2%) were of female mosquitoes, 8.5% were males, and 0.3% included both male and female mosquitoes. Most IAS were identified as *Aedes albopictus* (1582 confirmed, 315 suspected), followed by *Aedes japonicus* (373 confirmed, 54 suspected), *Aedes koreicus* (58 confirmed, 1 suspected), and *Aedes aegypti* (16 confirmed, 3 suspected). A further 25 mosquito observations were classified as *Aedes japonicus*/*koreicus.*

iNaturalist community-generated identification was found to be accurate for 87.6% (1386/1582) of expert-identified *Ae. albopictus* observations, 81.3% (13/16) for *Ae. aegypti* observations, and 60.3% (275/456) for combined *Ae. japonicus* and *Ae. koreicus* observations. An additional 92 observations of native mosquitoes were misidentified as IAS by iNaturalist users.

### 3.2. Aedes aegypti

*Aedes aegypti* is not widely established in Europe and neighbouring areas, with populations as of October 2023 known in Cyprus; Egypt; Madeira, Portugal; and the Black Sea coast region of Russia, Georgia, and Turkey [30]. The mosquito is also recorded as ‘introduced’ on the Canary Islands, Spain. Sixteen iNaturalist mosquito observations were identified as *Ae. aegypti*, and a further three were classified as ‘suspected *Ae. aegypti’.* Most of these were recorded on Madeira (15 confirmed and 3 suspected), whilst an additional observation was recorded on Tenerife, Canary Islands.

### 3.3. Aedes albopictus

**Figure 2:**
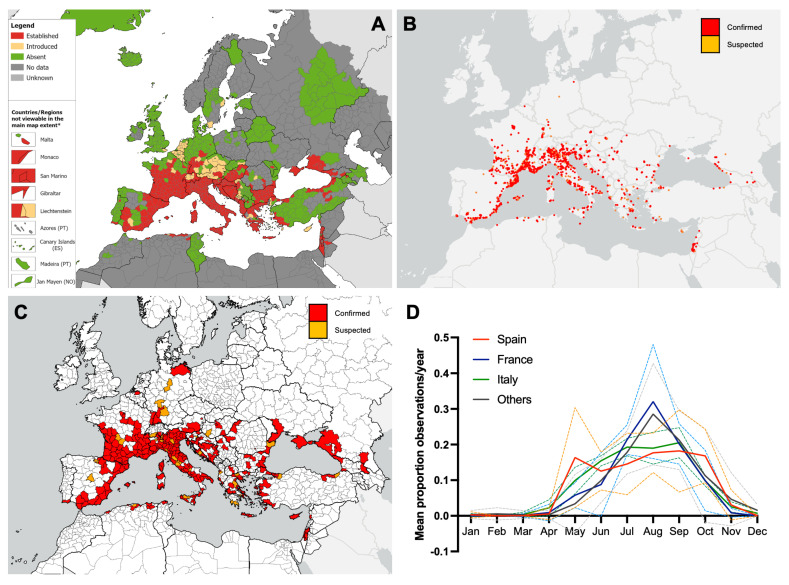
(**A**) Recorded distribution of *Aedes albopictus* in Europe and neighbouring countries as of October 2023 (ECDC/EFSA VectorNet project data). Distribution is shown at the ‘regional’ administrative unit level—NUTS3 or equivalent and NUTS2 in Austria, Belgium, Denmark, Germany, The Netherlands, and the United Kingdom. * Countries/regions are shown at different scales to facilitate visualisation. The boundaries and names on this map do not imply official endorsement or acceptance by the European Union. (**B**,**C**) iNaturalist observations of *Aedes albopictus* in Europe and neighbouring countries to the end of 2023, coloured according to confirmed and suspected identifications. (**B**), point data. (**C**), at the NUTS2/3 level. (**D**) Seasonality of iNaturalist observations of *Aedes albopictus* (confirmed only) shown as the mean proportion of monthly observations per the year 2020–2023. The line shows the mean for the three countries with the most *Aedes albopictus* observations and the remaining countries combined. Dotted lines indicate error bars, matching the colour of the mean and showing 95% confidence intervals.

The Asian tiger mosquito is now widely established in southern Europe, found in most areas along the Mediterranean (Figure 2A), and more recently has expanded its distribution further northward into central and northern France and central Germany. As of October 2023, multiple introductions had also been detected in more northerly European countries, including Belgium, The Netherlands, Sweden, and the United Kingdom, while the mosquito’s distribution in Central and Eastern Europe is also expanding (Figure 2A). This distribution is also reflected in iNaturalist data, with observations concentrated in the Mediterranean areas of Italy, France, and Spain, as well as more scattered observations throughout the known range (Figure 2B). Confirmed iNaturalist observations of *Ae. albopictus* were recorded in 30 out of 36 (83.3%) countries with established populations and 2/9 (22.2%) countries with introductions (Figure 2C; Table 2). However, iNaturalist data typically covered fewer regions within countries than VectorNet data. In 10 countries, iNaturalist observations were found in regions without VectorNet records of *Ae. albopictus* (Figure 2C; Table 2). iNaturalist observations of *Ae. albopictus* were recorded from May to November, with peak observations occurring from July to September (Figure 2D). Higher activity was observed earlier in the season in Spain and Italy than in France and all other countries combined (Figure 2D).

### 3.4. Aedes japonicus

**Figure 3:**
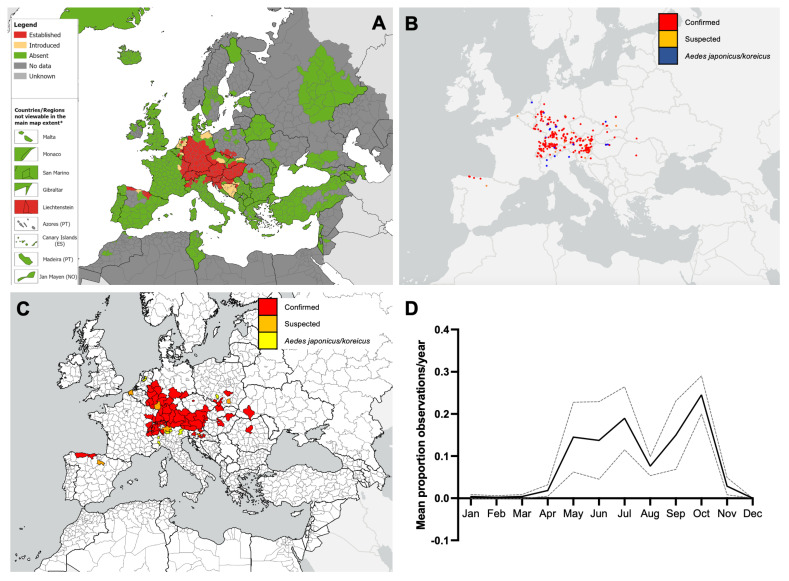
(**A**) Recorded distribution of *Aedes japonicus* in Europe and neighbouring countries as of October 2023 (ECDC/EFSA VectorNet project data). Distribution is shown at the ‘regional’ administrative unit level—NUTS3 or equivalent and NUTS2 in Austria, Belgium, Denmark, Germany, The Netherlands, and the United Kingdom. * Countries/regions are shown at different scales to facilitate visualisation. The boundaries and names on this map do not imply official endorsement or acceptance by the European Union. (**B**,**C**) iNaturalist observations of *Aedes japonicus* in Europe and neighbouring countries to the end of 2023. (**B**), point data. (**C**), at the NUTS2/3 level. (**D**) Seasonality of iNaturalist observations of *Aedes japonicus* (confirmed only) shown as the mean proportion of monthly observations per the year 2020–2023. The line shows the mean, and the error bars show the 95% confidence intervals.

As of October 2023, *Aedes japonicus* was established in 18 European countries, primarily in central Europe, and ‘introduced’ in one country (Figure 3A; Table 3). Observations of *Ae. japonicus* by iNaturalist users also show a similar geographic pattern (Figure 3B). Due to the similarity of *Ae. japonicus* and *Ae. koreicus*, some mosquito images could not be differentiated between the two species, although these were mostly within the known distribution of *Ae. japonicus* and are considered more likely to be this species (Figure 3B). Observations from iNaturalist included confirmed or suspected *Ae. japonicus* in 16/18 (88.9%) countries with established populations of this species (Figure 3C; Table 3), although regional coverage of each country was usually lower for iNaturalist data than for VectorNet data. iNaturalist observations of confirmed or suspected *Ae. japonicus* were also found in regions of six countries without VectorNet records and in one country (Ukraine) without VectorNet records (Figure 3C; Table 3). iNaturalist observations of *Ae. japonicus* began in April and lasted until November. There appeared to be two distinct peaks of activity, the first from May to July and the second from September to October (Figure 3D).

### 3.5. Aedes koreicus

**Figure 4:**
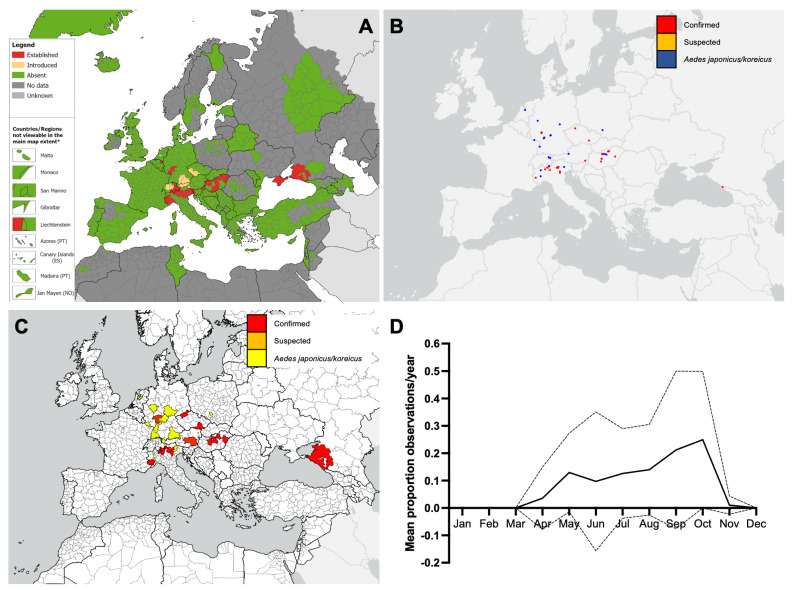
(**A**) Recorded distribution of *Aedes koreicus* in Europe and neighbouring countries as of October 2023 (ECDC/EFSA VectorNet project data). Distribution is shown at the ‘regional’ administrative unit level—NUTS3 or equivalent and NUTS2 in Austria, Belgium, Denmark, Germany, The Netherlands, and the United Kingdom. * Countries/regions are shown at different scales to facilitate visualisation. The boundaries and names on this map do not imply official endorsement or acceptance by the European Union. (**B**,**C**) iNaturalist observations of *Aedes koreicus* in Europe and neighbouring countries to the end of 2023. (**B**), point data. (**C**), at the NUTS2/3 level. (**D**) Seasonality of iNaturalist observations of *Aedes koreicus* (confirmed only) shown as the mean proportion of monthly observations per the year 2020–2023. The line shows the mean, and the error bars show the 95% confidence intervals.

The distribution of *Ae. koreicus* in Europe is less expansive than that of *Ae. albopictus* or *Ae. japonicus*, with nine countries known to harbour established populations and two countries that have detected introductions (Figure 4A; Table 4). Most iNaturalist observations of *Ae. koreicus* were in Hungary (30), Italy (19), and Germany (5), with additional records in Austria, Czechia, and Russia (Figure 4B). Most of the observations classified as *Aedes japonicus*/*koreicus* were considered more likely to be *Ae. japonicus* based on the wider distribution of this species, although those in Italy are considered more likely to be *Ae. koreicus.* The *Ae. japonicus*/*koreicus* observation in The Netherlands might be either species because both have been introduced to this country in the past. Confirmed iNaturalist observations of *Ae. koreicus* were identified in 6/11 (54.5%) countries with established or introduced *Ae. koreicus* (Figure 4C; Table 4). In Austria and Czechia, iNaturalist observations of *Ae. koreicus* were recorded in regions without VectorNet records of the species (Figure 4C; Table 4). Although only a low number of *Ae. koreicus* observations were made, they were made from April to November, with activity increasing over the season to peak in September and October (Figure 4D).

## 4. Discussion

Community-contributed observations of arthropod vector species on iNaturalist have previously been shown to be of value for monitoring the distribution, diversity, and seasonal occurrence of vectors encountered by humans [19,20,21,23]. This study presents further evidence that iNaturalist data can provide additional data on invasive mosquito species, which could be used in combination with locally targeted surveillance and other sources of mosquito data, such as Mosquito Alert, to identify new areas of introduction or geographic expansion. Numerous instances of IAS were identified from iNaturalist in areas without existing VectorNet records of introduction or establishment, highlighting the application of this approach. As an example of this in action, recently iNaturalist observations of *Ae. japonicus* were used in tandem with collected specimens to document the first records of this invasive species in Poland [31]. Similarly, the northward expansion of the lone star tick *Amblyomma americanum* into Michigan, United States, was accompanied by iNaturalist observations of this tick by the public in the same county [32]. Data from iNaturalist have also been successfully used to document the occurrence of numerous other invasive species [33,34,35,36,37,38,39,40].

While iNaturalist data gave a relatively good high-level (continent-scale) approximation of the distribution of these IAS in Europe, at a finer scale, this data source under-represented the known distributions of the mosquito species. This implies that locally targeted surveillance will be necessary to confirm and delineate the range of invasive species following the identification of potential new areas by iNaturalist data. A study using iNaturalist data to monitor the distribution of ticks across North America showed a similar result [21]—the data gave a good high-level representation of the known distributions of important tick vectors but was patchy at a local level, suggesting that iNaturalist would not be a good standalone surveillance system, but rather works best as a complement to other forms of vector surveillance. Likewise, a study investigating the effectiveness of iNaturalist data in characterising wildlife mass mortality events showed that although iNaturalist observations were useful for identifying the species, timing, and location of events, they underestimated their magnitude and full geographic scale [41]. Therefore, it was concluded that using iNaturalist to characterise wildlife mortality events was ineffective on its own, and best suited for supplementing conventional methods. Implementing a system combining data from multiple community science initiatives, such as the Global Mosquito Observations Dashboard, which combines mosquito data from iNaturalist, Mosquito Alert, and GLOBE Observer [22], to support and inform active surveillance may be the best approach for integrating iNaturalist data into ongoing vector surveillance.

iNaturalist observations were also examined to determine the seasonal occurrence of IAS, and data were found to concur with documented seasonal activity for these species [24]. For *Ae. albopictus*, the results also suggested regional differences in the activity of human-mosquito encounters, with activity in southern European countries (Spain and Italy) beginning earlier than in France, where the mosquito is also established in more northerly areas. Regional differences were also noted in iNaturalist tick observations in the United States [21], suggesting that iNaturalist data can be useful for delineating peak periods of risk for human-vector contact and, hence, disease transmission risk.

Overall, the use of iNaturalist data to support the surveillance of arthropod vectors has multiple benefits, including open access to real-time data and wide geographic coverage. However, this approach also suffers some drawbacks, some of which are unique to the platform and others common among passive surveillance methods. These are discussed in detail elsewhere [19,21,23] but include the bias of data collection to areas of high population density. This is evident in this study, where highly populated and more developed countries had higher numbers of mosquito observations and, therefore, a greater likelihood of detecting IAS. Another potential issue is the range of image quality associated with observations. The invasive mosquito species examined in this study have distinct characteristics that, in clear images, make them easily identifiable in comparison to most European species. However, this is made more difficult in blurry images, images of damaged mosquitoes, and in regions where similar mosquito species occur. For example, *Aedes cretinus*, which is present in Cyprus, Greece, and Turkey, has a similar appearance to *Ae. albopictus,* and a clear image of the scutum is required for confident separation [28]. Similarly, *Ae. japonicus* and *Ae. koreicus* were often difficult to separate due to requiring detailed imagery of hind tarsi for differential diagnosis. In this study, a simplified identification categorization of confirmed and suspected was used to separate observations with different levels of confidence in identification, but a more detailed and reproducible method of identification should be implemented in the future to reduce potential bias. One such algorithm, taking into account features required for species identification, clarity of imagery, and accuracy of georeferencing, has recently been proposed to score confidence in iNaturalist observations [42]. Although iNaturalist has its own observation quality grade, multiple studies have found that this is not an adequate measure of identification accuracy [23,42,43], and therefore, experienced researchers must verify iNaturalist data before use. This data validation by experts is also a vital component of existing IAS-focused citizen science programs such as Mosquito Alert [12]. In this study, all Culicidae records were downloaded, rather than just those identified as IAS, allowing the identification of additional IAS observations that were either unidentified or misidentified, but also served to document the number and geographic spread of mosquito observations across Europe. It was found that a considerable proportion of IAS observations in iNaturalist were not identified to species level or were incorrectly identified, and this was much higher (40%) for *Ae. japonicus*/*koreicus*, species that are less recognizable to the public compared to *Ae. albopictus* and *Ae. aegypti,* which have gained greater attention due to their higher public health importance. This suggests that searching the iNaturalist database only for species of interest is likely to exclude many records that have not yet been identified.

These results demonstrate the application of iNaturalist to support the monitoring of mosquito diversity and the introduction/expansion of IAS. This study provides further evidence that this platform is a cost-effective source of arthropod data that can assist existing vector surveillance, but it also suggests that expert validation of species identification is required to maximize the use of this data.

## Figures and Tables

**Table 1:** Number of iNaturalist observations of Culicidae and invasive *Aedes* species (IAS) in Europe and neighbouring countries. Countries where observations of IAS were identified are marked in bold.

Country	Culicidae Observations	Total IAS Observations (Confirmed/Suspected)	% IAS of All Mosquitoes (Confirmed (Including Suspected))
**Albania**	**53**	**4/4**	**7.5% (15.1%)**
**Algeria**	**68**	**14/2**	**20.6% (23.5%)**
Andorra	0	0	-
Armenia	1	0	0.0%
**Austria**	**1204**	**204/27**	**16.9% (19.2%)**
Azerbaijan	4	0	0.0%
Belarus	80	0	0.0%
**Belgium**	**197**	**1/1**	**0.5% (1.0%)**
**Bosnia & Herzegovina**	**11**	**6/0**	**54.5%**
**Bulgaria**	**67**	**17/3**	**25.4% (29.9%)**
**Croatia**	**136**	**50/4**	**36.8% (39.7%)**
**Cyprus**	**23**	**1/5**	**4.3% (26.1%)**
**Czechia**	**152**	**4/0**	**2.6%**
Denmark	408	0	0.0%
Egypt	18	0	0.0%
Estonia	33	0	0.0%
Faroe Islands	0	0	-
Finland	497	0	0.0%
**France** ^**a**^	**1906**	**487/103**	**25.6% (31.0%)**
**Georgia**	**23**	**7/3**	**30.4% (43.5%)**
**Germany**	**1700**	**147/18**	**8.6% (9.7%)**
**Gibraltar**	**2**	**1/0**	**50.0%**
**Greece**	**241**	**48/22**	**19.9% (29.0%)**
Greenland	20	0	0.0%
**Hungary**	**384**	**45/3**	**11.7% (12.5%)**
Iceland	0	0	-
Ireland	41	0	0.0%
**Israel**	**101**	**36/3**	**35.6% (38.6%)**
**Italy**	**1466**	**486/82**	**33.2% (38.7%)**
Jordan	5	0	0.0%
Kosovo	16	0	0.0%
Latvia	30	0	0.0%
**Lebanon**	**5**	**1/0**	**20.0%**
Libya	0	0	-
Liechtenstein	1	0	0.0%
Lithuania	125	0	0.0%
**Luxembourg**	**72**	**5/0**	**6.9%**
**Malta**	**10**	**2/0**	**20.0%**
Moldova	12	0	0.0%
Monaco	1	0	0.0%
**Montenegro**	**10**	**4/0**	**40.0%**
Morocco	17	0	0.0%
**The Netherlands**	**322**	**1/0**	**0.3%**
**North Macedonia**	**7**	**4/0**	**57.1%**
Norway	54	0	0.0%
**Palestinian Territories**	**6**	**1/0**	**16.7%**
**Poland**	**330**	**7/1**	**2.1% (2.4%)**
**Portugal ^b^**	**552**	**29/5**	**5.3% (6.2%)**
**Romania**	**67**	**17/2**	**25.4% (28.4%)**
**Russia ^c^**	**1801**	**15/3**	**0.8% (1.0%)**
San Marino	0	0	-
**Serbia**	**40**	**4/0**	**10.0%**
**Slovakia**	**41**	**1/0**	**2.4%**
**Slovenia**	**54**	**10/3**	**18.5% (24.1%)**
**Spain** ^**d**^	**1213**	**282/58**	**23.2% (28.0%)**
Sweden	259	0	0.0%
**Switzerland**	**269**	**84/14**	**31.2% (36.4%)**
**Syria**	**9**	**1/0**	**11.1%**
**Tunisia**	**7**	**1/0**	**14.3%**
**Turkey**	**108**	**24/7**	**22.2% (28.7%)**
**Ukraine**	**524**	**3/0**	**0.6%**
United Kingdom	1032	0	0.0%
*All countries*	**15,835**	**2054/373**	**13.0%/15.3%**

^a^ France includes Corsica. ^b^ Portugal includes Azores, Ilhas Selvagens and Madeira. ^c^ Russia, west of longitude 68. ^d^ Spain includes Ceuta, Melilla, the Balearic Islands, and the Canary Islands.

**Table 2:** *Aedes albopictus* detections by iNaturalist in countries with known populations, in comparison to VectorNet data (October 2023).

Country	*Aedes albopictus* Status VectorNet October 2023	No. Territories VectorNet Established/Introduced (Total Regions)	No. Territories with iNaturalist Observations Confirmed (Suspected)
Albania	established	12/0 (12)	2 (1)
Algeria	established	8/1 (58)	7 (1) *
Armenia	established	1/0 (11)	0
Austria	established	2/7 (9)	3
Belgium	*introduced*	0/9 (11)	1
Bulgaria	established	25/0 (28)	7
Bosnia & Herzegovina	established	3/0 (3)	2
Croatia	established	21/0 (21)	8 (1)
Cyprus	*introduced*	0/1 (1)	1
Czechia	*introduced*	0/3 (8)	0
France	established	71/2 (96)	51 (2)
Georgia	established	5/0 (12)	4 *
Germany	established	8/4 (38)	3 (3) *
Gibraltar	established	1/0 (1)	1
Greece	established	40/4 (52)	26 (5) *
Hungary	established	15/0 (20)	3 (1)
Israel	established	6/0 (6)	5
Italy	established	107/0 (107)	80 (10)
Jordan	established	4/0 (12)	0
Kosovo	established	1/0 (5)	0
Lebanon	established	8/0 (9)	1
Liechtenstein	*introduced*	0/1 (1)	0
Luxembourg	*introduced*	0/1 (1)	0
Malta	established	1/0 (1)	1
Moldova	established	1/0 (11)	0
Monaco	established	1/0 (1)	0
Montenegro	established	12/9 (25)	2
The Netherlands	*introduced*	0/9 (12)	0
North Macedonia	established	2/0 (8)	2 *
Palestine	established	2/0 (2)	1
Portugal	established	2/0 (25)	1
Romania	established	6/1 (42)	5 (1) *
Russia	established	2/0 (56)	1
San Marino	established	1/0 (1)	0
Serbia	established	3/5 (25)	2
Slovakia	*introduced*	0/2 (8)	0
Slovenia	established	9/0 (12)	3 (1)
Spain	established	27/1 (50)	19 (2)
Sweden	*introduced*	0/2 (21)	0
Switzerland	established	4/3 (7)	3
Syria	established	2/0 (14)	1 *
Tunisia	established	1/0 (24)	1 *
Turkey	established	16/1 (81)	8 (2) *
United Kingdom	*introduced*	0/2 (40)	0
Ukraine	established	2/0 (27)	2 *

* Indicates regional units with iNaturalist observations that are not represented in VectorNet data.

**Table 3:** *Aedes japonicus* detections by iNaturalist in countries with known populations, in comparison to VectorNet data (October 2023).

Country	*Aedes japonicus* Status VectorNet October 2023	No. Territories VectorNet Established/Introduced (Total Regions)	No. Territories with iNaturalist Observations Confirmed (Suspected)
Austria	established	9/0 (9)	9
Belgium	established	2/1 (11)	0 (1) *
Bosnia & Herzegovina	*introduced*	0/3 (3)	0
Croatia	established	14/2 (21)	1
Czechia	established	3/1 (8)	2
France	established	8/1 (96)	4 *
Germany	established	31/1 (38)	20 (1)
Hungary	established	20/0 (20)	2
Italy	established	17/0 (107)	0 (4)
Liechtenstein	established	1/0 (1)	0
Luxembourg	established	1/0 (1)	1
The Netherlands	established	2/6 (12)	0 (1)
Poland	established	11/0 (73)	5 (2) *
Romania	established	1/0 (42)	1 *
Serbia	established	1/1 (25)	0
Slovakia	established	3/1 (8)	1 *
Slovenia	established	12/0 (12)	2 (1)
Spain	established	5/1 (50)	2 (1) *
Switzerland	established	7/0 (7)	7
Ukraine	absent	0/0 (27)	1 *

* Indicates regional units with iNaturalist observations that are not represented in VectorNet data.

**Table 4:** *Aedes koreicus* detections by iNaturalist in countries with known populations, in comparison to VectorNet data (October 2023).

Country	*Aedes japonicus* Status VectorNet October 2023	No. Territories VectorNet Established/Introduced (Total Regions)	No. Territories with iNaturalist Observations Confirmed (Suspected)
Austria	established	1/1 (9)	1 *
Belgium	established	1/0 (11)	0
Czechia	*introduced*	0/1 (8)	2 *
Germany	established	2/2 (38)	1
Hungary	established	14/0 (20)	4
Italy	established	24/0 (107)	7 (3)
The Netherlands	*introduced*	0/1 (12)	0 (1) *
Russia	established	1/0 (56)	1
Slovenia	established	2/0 (12)	0
Switzerland	established	2/3 (7)	0
Ukraine	established	2/0 (27)	0

* Indicates regional units with iNaturalist observations that are not represented in VectorNet data.

## Data Availability

The original contributions presented in this study are included in the article/Supplementary Materials. Further inquiries can be directed to the corresponding author.

## Supplementary Materials

The following supporting information can be downloaded at https://www.mdpi.com/article/10.3390/insects16020128/s1, Table S1: Raw cleaned data of iNaturalist Culicidae observations used in this study; Table S2: Summary of Culicidae observations and invasive *Aedes* species identified in each country.
